# Principles of mechanical and chemical debridement with implant retention

**DOI:** 10.1186/s42836-023-00170-x

**Published:** 2023-04-06

**Authors:** David G. Deckey, Zachary K. Christopher, Joshua S. Bingham, Mark J. Spangehl

**Affiliations:** Department of Orthopaedic Surgery, Mayo Clinic, Phoenix, AZ 85054 USA

**Keywords:** Prosthetic joint infection, DAIR, Debridement, Irrigation, Periprosthetic joint infection, Implant retention, Acute infection

## Abstract

**Background:**

Periprosthetic joint infection (PJI) is one of the most common causes of early revision for total hip and knee arthroplasty. Mechanical and chemical debridement typically referred to as debridement, antibiotics, and implant retention (DAIR) can be a successful technique to eradicate PJI in acute postoperative or acute hematogenous infections. This review will focus specifically on the indications, techniques, and outcomes of DAIR.

**Discussion:**

The success of mechanical and chemical debridement, or a DAIR operation, is reliant on a combination of appropriate patient selection and meticulous technique. There are many technical considerations to take into consideration. One of the most important factors in the success of the DAIR procedure is the adequacy of mechanical debridement. Techniques are surgeon-specific and perhaps contribute to the large variability in the literature on the success of DAIR. Factors that have been shown to be associated with success include the exchange of modular components, performing the procedure within seven days or less of symptom onset, and possibly adjunctive rifampin or fluoroquinolone therapy, though this remains controversial. Factors that have been associated with failure include rheumatoid arthritis, age greater than 80 years, male sex, chronic renal failure, liver cirrhosis, and chronic obstructive pulmonary disease.

**Conclusions:**

DAIR is an effective treatment option for the management of an acute postoperative or hematogenous PJI in the appropriately selected patient with well-fixed implants.

## Background

Periprosthetic joint infection (PJI) remains one of the most common causes of early revision surgery for total hip and knee arthroplasty (THA, TKA), particularly within three months of the index surgery [1]. PJI is not only costly to the global healthcare system but it is also associated with significant morbidity to patients, even when successfully treated [2, 3]. Success rates can be variable based on a multitude of factors, including treatment technique [4]. While there are several different surgical options for the management of PJI, this review will focus specifically on the indications, techniques, and outcomes of mechanical and chemical debridement, commonly referred to as debridement, antibiotics, and implant retention (DAIR).

## Indications & timing

The management of PJI varies based on a variety of factors such as host comorbidities, fixation and functional status of the implants, infecting organisms, and commonly the chronicity of the infection. For chronic infections, one-stage or two-stage revision remains the gold standard. DAIR procedures are less morbid than formal two-stage reimplantation procedures [5], yet their success can be variable [6–16]. DAIR should be considered for acute postoperative infections or acute hematogenous infections, defined as symptoms existing for no longer than 4 weeks, with stable and well-functioning implants.

While an acute PJI is not a surgical emergency, it should be dealt with urgently once the patient is medically optimized. Narayanan et al*.* demonstrated that patients with early PJI after TKA treated with DAIR less than two weeks after surgery had higher rates of success compared with those treated two weeks after the index procedure [14]. Additionally, the identification of pathogens preoperatively is recommended. Certain pathogens, such as *Pseudomonas aeruginosa* and methicillin-resistant *Staphylococcus aureus* (MRSA), are associated with higher failure rates with DAIR, and thus preoperative cultures should also be obtained [5]. Tarity et al*.* argue that the microbial species may be more clinically relevant to the success of DAIR than even chronicity [15]. Additionally, identification of the organism preoperatively may influence antibiotics used if antibiotic-loaded cement beads are used intraoperatively. Next-generation sequencing (NGS) has been proposed as a possible adjunct to more rapidly identify pathogens or identify pathogens in a culture-negative setting [17–19]. Future studies are needed to further explore the role of NGS in PJI.

## Technical aspects of the procedure

### Overview aspects of the procedure

DAIR can be a successful technique to eradicate PJI in the appropriately selected patient. After patient selection, one of the keys to a successful DAIR procedure is attention to detail, specifically to the technical components of the procedure [8, 20, 21]. At the authors' institution, the prior incision is most frequently utilized. DAIR requires thorough debridement with a meticulous technique to maximize bacterial bioburden eradication. Multiple tissue samples are taken and sent for bacterial and fungal cultures. The integrity of implants should be assessed to ensure fixation to bone. Irrigation can be augmented with various antiseptics or antibiotics, but mechanical debridement, with a meticulous synovectomy and soft tissue debridement along with the exchange of modular components, when possible, remains the critical component of the procedure. A thorough mechanical debridement can take time and should not be rushed.

### Changing drapes

Prior to the start of the procedure, the surgeon must consider the room setup. To limit bacterial burden, surgeons may choose to have a separate “clean” and “dirty” setup on separate back tables. All drapes, gowns, gloves, and instruments should be exchanged for clean ones after the debridement and irrigation, and prior to the implantation of the new modular components. This theoretically decreases contamination from the infected surgical site. The impact of changing drapes during DAIR has not been directly investigated and therefore may be left to the surgeon's discretion [22]. Although this has not been proven, the application of clean drapes after the irrigation and debridement portion of the surgery, such as the “Double Draping” technique described by Melnic, would seem to be a prudent decision [23].

### Exchange of modular components

Exchanging modular components for new ones is an important factor in reducing bacterial load. Not only does exchanging polyethylene components or a modular head remove any biofilm on that material, but it also allows for better access to otherwise difficult-to-reach areas of the joint. Removing the polyethylene in a TKA provides improved visualization into the posterior aspect of the knee for a complete synovectomy. Improved visualization will allow for a more thorough debridement overall. Several studies supported the exchange of modular components as one method to reduce the rate of PJI recurrence [4, 11, 16, 24, 25]. A meta-analysis of 39 retrospective studies demonstrated a higher success rate (73.9% *vs.* 60.7%) in THA treated for PJI who underwent exchange of modular components [16]. Another multicenter study demonstrated a 33% decrease in failure rates with modular component exchange [24]. Overall, the success rate of the DAIR procedure is quite variable, but this is one factor that appears to decrease the risk of failure and should be performed. The International Consensus Meeting (ICM) on Orthopedic Infections recommends modular component exchange with moderate strength of evidence [22].

### Mechanical debridement

The adequacy of mechanical debridement is perhaps the most important factor in the success of the DAIR procedure, but arguably the hardest to study. Techniques are surgeon-specific and perhaps contribute to the large variability in the literature of the success of DAIR. Regardless, removal of all devitalized and infected tissues as well as inflamed and hypertrophic synovium is recommended. A radical synovectomy may be performed to eliminate all infected tissue around the joint. At times it may be difficult to distinguish between infected and non-infected tissue. Usually, a tissue plane can be developed between more normal tissue and the infected and inflamed synovium. In principle, all non-vital tissue should be debrided including synovium and thickened scar tissue. Thus, a radical synovectomy is performed and in TKA’s, careful attention is given to the suprapatellar pouch and medial and lateral gutters. The posterior aspect of the knee can be debrided using a rougher to remove thickened, inflamed synovium. Additionally, the deep aspect of the extensor mechanism should also be debrided, along with resection of the soft tissue “meniscus” namely the thick layer of soft tissue that typically forms around the periphery of the patellar implant. All retained non-modular components should be thoroughly scrubbed with a sterile brush and then irrigated with an antimicrobial/antiseptic solution and sterile saline.

### Irrigation

The ideal irrigation type, volume, and pressure have not been established. Sterile saline irrigation is ubiquitous throughout surgery to mechanically remove contaminants from the surgical field. However, there are no clinical studies evaluating the optimal volume of irrigation during DAIR for the treatment of a PJI. One study evaluated the volume necessary to eliminate particles of cement and bone less than 1 μm in size, and recommended 4 L of irrigation. Extrapolating these results, bacteria of this size may similarly be removed with approximately 4 L of irrigation. The ICM gave a strong consensus recommendation by the super majority that 6–9 L of irrigation appears to be sufficient [22].

Furthermore, the pressure of irrigation has not been well-defined. There is no strong clinical evidence supporting the use of low- or high-pressure irrigation specifically. In the landmark Fluid Lavage of Open Wounds (FLOW) trial, investigators found no difference between high or low-pressure irrigation with sterile saline in open fracture care [26]. Other studies have suggested that high-pressure lavage may propagate contaminants further into the wound in open fractures [27]. Low-pressure lavage may be sufficient for the prevention of infection during primary arthroplasty, but not be as effective in the setting of the high bacterial loads of a PJI. There is no current literature that supports the use of one method over the other. A randomized control trial compared high- and low-pressure lavage in PJI and found no difference in reinfections at 1-year follow-up [28].

### Antiseptic irrigation solutions

Antiseptic irrigation solutions have emerged as a tool to aid in reducing the risk of infection in aseptic arthroplasty surgeries. Most of the current arthroplasty literature focuses on dilute povidone-iodine and chlorhexidine in the setting of primary and revision total joint arthroplasty. A recent critical analysis review outlined common antiseptics used in TJA and the most current evidence [29]. The authors proposed that irrigation with antiseptic solutions may decrease infection risk when used prophylactically in TJA with a grade B strength of recommendation. However, they also concluded that there is insufficient evidence to recommend a gold-standard solution.

In the setting of a DAIR procedure, there is also insufficient evidence to recommend the use of a specific antiseptic or combination of antiseptics, yet basic principles would suggest that an antiseptic may be of benefit. Furthermore, given the profound consequences of a failed DAIR, it is reasonable to use a relatively low-cost, low-risk adjunct solution(s) to help eliminate the bacterial load.

Dilute povidone-iodine has been described as a safe and potentially beneficial irrigation solution [30]. In the setting of known infection, the presence of biofilm is one of the primary considerations. The efficacy of several antiseptic solutions at eliminating bacteria in biofilms has been studied, and the authors of a recent manuscript found povidone-iodine to be the most effective at eliminating MRSA in biofilms and acetic acid solutions to be more effective on several other organisms [31]. In vitro studies suggest that many antiseptic solutions including povidone-iodine, chlorhexidine, hydrogen peroxide, NaOCl, and acetic acid may be useful in the management of PJI [32–34]. There may be some benefit to using multiple solutions to treat known PJI in a DAIR procedure based on the properties of each agent. Surgeons should be aware, however, that combining multiple irrigation solutions has the potential to produce toxic by-products and damage to the soft tissue [35]. If multiple solutions are used, saline lavage between various agents should be performed. Finally, many antiseptic solutions are not FDA-approved for intra-wound irrigation and further research is required on their use in DAIR.

### Methylene blue

Methylene blue is a cationic dye that can bind to eukaryotic cells and bacterial biofilms. Upon binding, the dye will stain these structures and can be a useful tool in guiding debridement of biofilms. In vitro models have demonstrated successful staining of *Staphylococcus epidermis* biofilms on orthopedic implants [36]. More recently, a study on the clinical application of methylene blue has demonstrated improved identification of staphylococcal biofilms in a comparison of stained *vs*. unstained tissue in cases of PJI [37]. In this study, methylene blue was diluted to a concentration of 0.1% and instilled for 60 s over the surgical site after the removal of the polyethylene. This may be a useful adjunct in DAIR to maximize the eradication of biofilms.

### Intraosseous antibiotics

Intraosseous (IO) administration of antibiotics should be considered in a DAIR procedure when treating infected total knee arthroplasties. IO vancomycin was initially studied in the setting of primary and revision TKA prophylaxis. Studies have demonstrated that IO vancomycin administration achieves dramatically higher tissue concentrations than intravenous administration in primary TKAs [38], revision TKAs [39], and even in cases of limited tourniquet usage [40]. IO vancomycin has the proposed benefit of higher tissue concentrations with lower doses, avoiding long infusion times, and minimizing systemic toxicity. It has also been shown to be effective in patients with BMI > 35, resulting in tissue concentration 5–9 times higher than systematic administration [41]. Recently, Kildow et al*.* published their results of DAIR supplemented with the use of IO vancomycin and noted high success rates (92.3% non-recurrence rate) in acute infections, but low success in chronic infections (44.4%) [12]. Administering IO antibiotics can be performed relatively quickly and safely into the proximal tibia. Current protocols recommend infusing 500 g of vancomycin diluted with 140 mL of normal saline into the proximal tibia over a period of 1–2 min after inflation of a thigh tourniquet [42].

### Double DAIR

A modification to the traditional single-stage DAIR has been also been described [20, 43, 44]. This planned, two-stage debridement, also known as the Double DAIR, has been shown to be generally more effective and more cost-effective, due to improved results, than a single DAIR [45]. This technique involves extensive mechanical debridement, removal/cleaning/reinsertion of modular components, thorough irrigation, and placement of high-dose antibiotic beads into the periprosthetic space during the first stage. The second stage, typically performed 5–7 days later, consists of a second debridement and irrigation, removal of antibiotic beads, and placement of new modular components. Even with the increased hospital length of stay and added procedure, Antonios et al*.* reported that the Double DAIR is more cost-effective than a single DAIR from a societal perspective. Chung et al. reported an overall success rate of 86.7% (72/82) in both THA and TKA with this technique at an average follow-up of 42 months [43]. This improved to 93.8% (45/48) when only primary arthroplasties were included.

## Postoperative considerations

### Antibiotic therapy

There is a large variation in the literature regarding the length of treatment, route of administration, and type of antimicrobial therapy for patients after DAIR. An algorithm proposed by Zimmerli and Ochsner consisting of 7 to 14 days of intravenous antibiotics, followed by 3 to 6 months of oral antibiotics directed against the bacteria in biofilms, has been cited widely throughout the literature [46]. Antibiotic therapy should be tailored based on intraoperative cultures and susceptibilities. Empiric treatment may be initiated once cultures are taken and should be based on the patient’s risk factors and infection history as well as the hospital's antibiogram. The duration and type of antimicrobial therapy depend on the organism. In general, 4–6 weeks of antibiotic therapy is recommended but can vary based on the specific organism and virulence. IV antibiotics should be administered for the first 10–14 days, followed by a transition to oral antibiotics for the remainder of the therapy according to the International Consensus Meeting guidelines. Co-management with an infectious disease specialist to help dictate antibiotic therapy and monitor appropriate dosing and laboratory markers is recommended. The ICM gives a moderate strength recommendation for a minimum of 6 weeks of antibiotic therapy after DAIR [47].

### Outcomes

There are several factors that have been associated with successful treatment of acute PJI with DAIR (Table 1). The exchange of modular components, particularly polyethylene, has been shown to increase the success rate of DAIR [16, 48]. Previously, studies have shown that bacteria adhere to materials with rougher surface microtopography, such as polyethylene [49–51]. The timing of surgical debridement has been shown to result in greater success if done within seven days or less from the onset of symptoms [7, 25, 52, 53]. After the procedure, the addition of rifampin in staphylococcal PJI [11, 24, 46, 54] or fluoroquinolones in Gram-negative PJI [55–57] has been supported. However, a recent systematic review did not find a benefit in improving treatment failure rates with adjunctive rifampin therapy after DAIR procedures [58]. Hence the benefit of adding rifampin for staphylococcal infections remains controversial.

**Table 1:** Factors associated with success and failure after DAIR

Factors Associated with Successful DAIR	Factors Associated with Failure of DAIR
Exchange of modular components	Host factors: rheumatoid arthritis, older age, male sex, chronic renal failure, liver cirrhosis, and chronic obstructive pulmonary disease
Performing the debridement within 7 days after onset of symptoms	Prosthesis for the treatment of fractures, cemented prostheses, and revision prostheses
The addition of rifampin to the antibiotic regimen	Highly elevated C-reactive protein on presentation
The treatment of Gram-negative bacilli with fluoroquinolones when indicated	PJI by *Staphylococcus aureus* or *Enterococcoci spp*.

Risk factors for the failure of DAIR have also been extensively studied (Table 1). There are certain host-related factors that are important to consider prior to performing DAIR. Rheumatoid arthritis has been shown to be a risk factor for failure of DAIR [24, 52, 59–61]. Age is associated with worse outcomes, especially in patients older than 80 years [62]. This same study showed a higher rate of failure in male sex as well. Chronic renal failure [57, 59, 63], liver cirrhosis [63, 64], and chronic obstructive pulmonary disease [65] are also associated with higher rates of failure. Naturally, given the relative immunocompromising nature of these co-morbidities, it makes intuitive sense that individuals with these conditions would be at a higher risk of failure. When a patient presents with significantly elevated inflammatory markers, such as a C-Reactive Protein > 115 mg/L, there is also a significantly higher rate of failure [24, 56, 62, 63, 66]. Moreover, patients with acute PJI in the setting of arthroplasty for the treatment of fracture or revision arthroplasty have higher failure rates after DAIR [67, 68]. Finally, certain pathogens portend a worse prognosis. *Staphylococcus aureus* [62, 69–71] and *Enterococci spp.* [71–74] have higher failure rates than other pathogens.

### Failure and treatment options

Overall, after treatment failure of a DAIR, a two-stage revision is often the next logical choice. Based on currently available evidence, DAIR does not appear to compromise the results of a subsequent two-stage exchange. Earlier studies suggested higher failure rates of a two-stage exchange after DAIR, however, subsequent studies do not demonstrate an increase in failure rates. Sherrell et al. reported that 34% of patients with prior DAIR failed subsequent two-stage revision for infection, but there was no comparison group [75]. A somewhat more recent retrospective review also reported a 24% failure of two-stage exchange following prior failed DAIR compared to a 16% failure rate after direct two-stage revision [76]. Contradicting the prior two studies, Brimmo et al. reported that the failure rate of a two-stage revision TKA is not increased by prior failed DAIR [77]. Nodzo et al*.* also reported near-identical success rates (82.2% *vs.* 82.5%) between two-stage revision arthroplasty after failed DAIR and isolated two-stage revision [78]. Finally, Kim and colleagues also suggested that prior failed DAIR does not compromise the success rate of a subsequent staged revision [79].

### Special circumstances

A unique group where DAIR can additionally be considered is in the treatment of acute or chronic PJI with extensive implant instrumentation. In select cases, patients may have such extensive instrumentation that removal of the implants would compromise limb function or risk amputation. In these scenarios, DAIR may be considered in addition to chronic, suppressive antibiotic therapy. Barry et al*.* showed that DAIR with chronic antibiotic suppression was as effective as two-stage revision in preventing reoperation for infection [80]. In fact, compared to the two-stage exchange group, the I&D and suppression group had more patients remaining ambulatory at final follow-up and more patients with functional knee range of motion.

### Recent innovation and future directions

Over the past 20 years, incremental improvements in the management of PJI have occurred [81, 82]. Although there is insufficient evidence to support which improvements have been the most efficacious, the authors believe that the biggest improvement has been the recognition of the importance of the meticulous technique of debridement. In addition, the use of antimicrobial irrigation has become more prevalent and may also decrease infection risk, although evidence is mixed. The introduction of intraosseous vancomycin has also been a substantial innovation [38, 39, 83]. This has been shown to be safe and effective at providing antibiotic prophylaxis while limiting the systemic side effects of vancomycin and emerging evidence shows a reduction in PJI. This reduction in PJI may also translate into improved management of acute knee PJI, though the data are still lacking. Lastly, a more novel technique of a “Double DAIR” has helped improve our ability to more effectively address the limitations of a single-stage DAIR and has shown encouraging results in successful infection management [20, 43, 44]. Going forward, a multimodal approach to infection prevention in addition to preoperative optimization will continue to be essential in preventing PJI.

## Conclusion

DAIR is an effective treatment option for the management of an acute postoperative or hematogenous PJI in appropriately selected patients with well-fixed implants. There are many considerations the treating surgeon should be aware of including preoperative factors, the need for a meticulous intraoperative debridement technique, and appropriate postoperative antibiotic management.

## Data Availability

Data sharing is not applicable to this article as no datasets were generated or analysed during the current study.

## Abbreviations

PJI: Periprosthetic joint infection
DAIR: Debridement, antibiotics and implant retention
TKA: Total knee arthroplasty
THA: Total hip arthroplasty
TJA: Total joint arthroplasty
MRSA: Methicillin-resistant *Staphylococcus aureus*
I & D: Irrigation and debridement
ICM: International Consensus Meeting
IO: Intraosseous
